# Yes, one can obtain better quality structures from routine X-ray data collection

**DOI:** 10.1107/S2052252515020941

**Published:** 2016-01-01

**Authors:** W. Fabiola Sanjuan-Szklarz, Anna A. Hoser, Matthias Gutmann, Anders Østergaard Madsen, Krzysztof Woźniak

**Affiliations:** a Biological and Chemical Research Centre, Chemistry Department, University of Warsaw, Żwirki i Wigury 101, 02-089 Warszawa, Poland; b Rutherford Appleton Laboratory, ISIS Facility, Chilton, Didcot, Oxfordshire OX11 OQX, UK; c Department of Chemistry, University of Copenhagen, Universitetsparken 5, DK-2100 Copenhagen, Denmark

**Keywords:** X-ray diffraction results, precision, independent atom model, transferable aspherical atom model, geometric parameters, TLS analysis

## Abstract

Single-crystal X-ray diffraction structural results for benzidine dihydrochloride, hydrated and protonated *N*,*N*,*N*,*N*-*peri*(dimethylamino)naphthalene chloride, triptycene, dichlorodimethyltriptycene and decamethylferrocene have been analysed. A critical discussion of the dependence of structural and thermal parameters on resolution for these compounds is presented. Results of refinements against X-ray data, cut off to different resolutions from the high-resolution data files, are compared to structural models derived from neutron diffraction experiments. The Independent Atom Model (IAM) and the Transferable Aspherical Atom Model (TAAM) are tested. The average differences between the X-ray and neutron structural parameters (with the exception of valence angles defined by H atoms) decrease with the increasing 2θ_max_ angle. The scale of differences between X-ray and neutron geometrical parameters can be significantly reduced when data are collected to the higher, than commonly used, 2θ_max_ diffraction angles (for Mo *K*α 2θ_max_ > 65°). The final structural and thermal parameters obtained for the studied compounds using TAAM refinement are in better agreement with the neutron values than the IAM results for all resolutions and all compounds. By using TAAM, it is still possible to obtain accurate results even from low-resolution X-ray data. This is particularly important as TAAM is easy to apply and can routinely be used to improve the quality of structural investigations [Dominiak (2015 ▸). *LSDB* from *UBDB.* University of Buffalo, USA]. We can recommend that, in order to obtain more adequate (more accurate and precise) structural and displacement parameters during the IAM model refinement, data should be collected up to the larger diffraction angles, at least, for Mo *K*α radiation to 2θ_max_ = 65° (sin θ_max_/λ < 0.75 Å^−1^). The TAAM approach is a very good option to obtain more adequate results even using data collected to the lower 2θ_max_ angles. Also the results of translation–libration–screw (TLS) analysis and vibrational entropy values are more reliable for 2θ_max_ > 65°.

## Abstract 2

Single-crystal X-ray diffraction data should be collected to the highest resolution as this allows for refinement of more reliable structural, thermal and dependent parameters. The results of refinements using a Transferable Aspherical Atomic Model of electron density (TAAM) appear to be in far better agreement with neutron results than the corresponding Independent Atom Model (IAM) results for all parameters, all resolutions and all compounds, and we advocate the use of this approach instead of IAM.

## 1. Introduction   

Single-crystal X-ray and neutron diffraction techniques are the most common experimental methods for obtaining detailed information about the three-dimensional structure of molecules in the crystalline state. Approximately 100 000 crystal structures of organic and inorganic compounds are determined each year using X-rays alone. Structural data is considered to be extremely useful in crystal chemistry, pharmacy, crystal engineering, materials science *etc.*, and is stored in the crystal structure databases such as the Cambridge Structural Database (CSD; Allen, 2002 ▸) or the Inorganic Crystal Structure Database (ICSD; Bergerhoff & Brown, 1987 ▸; Belsky *et al.*, 2002 ▸). Such data is commonly used in pharmaceutical, medical, biological and physicochemical studies or theoretical simulations. Macromolecular/protein single-crystal X-ray structural data is also compiled in the Protein Data Bank (Berman *et al.*, 2000 ▸). Wide applications of high-quality structural data are crucial for the further development of science as they are used to estimate the energy of inter- and intramolecular interactions. As small changes in geometrical parameters of molecules in crystals can lead to significant changes in conformational energy, it is important to identify not only which structural parameters undergo changes but also to estimate the magnitude of such changes.

In the case of X-ray diffraction, the quality of the final results of structural studies depends on several factors. One of the most important ones is the maximum diffraction angle, θ_max_ (or 2θ_max_), up to which the measured reflections are still taken into consideration during structure refinement. According to the IUCr Commission Guidelines (IUCr, 2012 ▸), the maximum diffraction angle of the measured reflections (θ_max_) for a single-crystal X-ray diffraction experiment intended for publication in crystallographic journals and crystallographic databases (CSD, ICSD) should be such that (sin θ/λ)_max_ exceeds 0.6 Å^−1^ (*i.e.* θ_max_ > 25° for Mo *K*α; θ_max_ > 67° for Cu *K*α).

An electron density model used in the refinement procedure is another important factor which has a crucial influence on the structural parameters obtained from an X-ray diffraction experiment. The simplest and most frequently applied model in modern structural crystallography is the Independent Atom Model (IAM) which assumes that the molecular electron density is the sum over spherical, non-interacting atoms. It was introduced by Bragg and Compton in the Max von Laue and the Braggs era. In fact, the first spherical atomic scattering factors were calculated by Hartree in 1925 (Hartree, 1925 ▸).

In more advanced models, the asphericity of the atoms is considered. These models were first introduced by Hirshfeld (1971 ▸) and later developed by Stewart (1976 ▸) and Hansen & Coppens (1978 ▸). In the Stewart and Hansen–Coppens models, the total atomic density is the sum over so-called pseudoatoms, and the electron density of each pseudoatom is given by the sum of three components (core, valence and valence deformation density). These models can be used only for the high-resolution X-ray diffraction data. As electronic parameters of the same type of atoms in identical topological environment appear to be grouped close to their average values, the idea of constructing databanks of pseudoatoms (the smallest atomic fragments of electron density), from which the full electron density distribution can be reconstructed, emerged (Brock *et al.*, 1991 ▸). There are three pseudoatom databanks: [UBDB (Koritsanszky *et al.*, 2002 ▸; Volkov *et al.*, 2004 ▸; Dominiak *et al.*, 2007 ▸; Jarzembska & Dominiak, 2012 ▸), Invariom (Dittrich *et al.*, 2004 ▸; Dittrich, Hubschle *et al.*, 2006 ▸; Hübschle *et al.*, 2007 ▸) and ELMAM (Pichon-Pesme *et al.*, 1995 ▸; Domagała *et al.*, 2012 ▸)]. ELMAM is based on purely experimental charge densities resulting from multipole refinement against high-resolution X-ray diffraction data, whereas the other two databases are based on theoretical results. Each of them can be applied in order to conduct Transferred Aspherical Atom Model (TAAM) refinement (Pichon-Pesme *et al.*, 1995 ▸; Dominiak *et al.*, 2007 ▸). A careful comparison of all databases has been discussed by Pichon-Pesme *et al.* (2004 ▸) and Bąk *et al.* (2011 ▸). With all these databases, it is possible to model electron density (ED) apparently better than by using IAM, and more accurately deconvolute thermal motion within TAAM refinement (Pichon-Pesme *et al.*, 1995 ▸; Volkov *et al.*, 2007 ▸). In TAAM refinement, pseudoatom parameters for each species are transferred from a chosen database and only atomic coordinates and ADPs are refined. Structural parameters obtained for the same X-ray data set after IAM and TAAM refinements are not the same.

We have decided to use the current version of the UBDB databank, *i.e.* UBDB2011 (Jarzembska & Dominiak, 2012 ▸). In UBDB, each atom type results from averaging electron density parameters over a family of chemically unique pseudo­atoms derived from the theoretical densities of a number of small molecules. The theoretical densities are obtained from B3LYP/6-31G** single-point calculations on the basis of experimental geometries taken from the CSD (Allen, 2002 ▸). In UBDB, only the valence structure factors are applied and the core electrons are added after the fitting procedure.

Neutron radiation is scattered by atomic nuclei. In consequence, H-atom positions and their displacement parameters can be determined more accurately using neutron diffraction than by applying single-crystal X-ray radiation. However, single-crystal neutron diffraction is less commonly used because of poor availability of the neutron sources and the required size of crystals. Although the most modern neutron facilities can provide reasonable results even for the sub­millimeter size single crystals, only *ca* 0.3% of all crystal structures added yearly to the structural databases are determined by neutron diffraction. Hereafter, we will abbreviate the geometry obtained from refinement of single-crystal neutron diffraction data by the term ‘neutron geometry’ written without quotation marks.

It was shown that a molecular geometry very close to the neutron geometry can be obtained after multipole refinement of high-resolution X-ray data (Hoser *et al.*, 2009 ▸). Moreover, it was shown that the TAAM refinement against high-resolution X-ray data significantly improves the molecular geometry (Dittrich, Munshi *et al.*, 2006 ▸; Hübschle *et al.*, 2007 ▸; Volkov *et al.*, 2007 ▸; Jelsch *et al.*, 2005 ▸) with respect to the independent atom model (IAM) and also leads to ADPs closer to those obtained from multipole refinements (Volkov *et al.*, 2007 ▸; Dittrich *et al.*, 2008 ▸; Bąk *et al.*, 2009 ▸). Additionally, the results of TAAM refinement appear to give molecular geometries in excellent agreement with optimized geometries from *CRYSTAL*09 (Dovesi *et al.*, 2005 ▸) calculations (Jarzembska *et al.*, 2012 ▸). Despite the fact that TAAM significantly improves the model for high-resolution data, there were also some reports which show that possibly it should also give reasonable models for low-resolution data (Bąk *et al.*, 2011 ▸). However, the use of TAAM refinement has not gained much attention yet.

Therefore, we would like to investigate how geometrical parameters change for different maximum θ_max_ diffraction angles in the case of IAM and TAAM refinements. Such an analysis may indicate how similar these models can get to the neutron geometry, especially when it was only possible to measure the low-resolution data. We have focused on the comparison of the structural neutron and X-ray results for crystals of five model compounds of different complexity and quality of data in the case of IAM, and for three of them in the case of the TAAM model. We also would like to verify whether commonly used resolution limits of data allow the best atomic geometrical and thermal parameters to be obtained.

Our work also includes an analysis of thermal parameters which are obtained after the IAM and TAAM refinements against X-ray diffraction data cut to different θ_max_ values. When ADPs are good quality, it is possible to conduct a TLS analysis (Cruickshank, 1956 ▸; Schomaker & Trueblood, 1968 ▸) and derive frequencies for translational and librational normal modes. Recently it was shown by Madsen & Larsen (2007 ▸) that such frequencies can be utilized to estimate the vibrational entropy of a crystal. Analysis of thermal motion conducted by Madsen & Larsen (2007 ▸) rely on ADPs obtained from high-resolution X-ray data for xylitol and ribitol. Lately it was suggested by Jarzembska *et al.* (2014 ▸) that high-resolution data are not necessarily needed and that to estimate vibrational entropy it is enough to conduct TAAM refinement on a low-resolution data. In our study we would like to verify this possibility.

**Figure 1:**
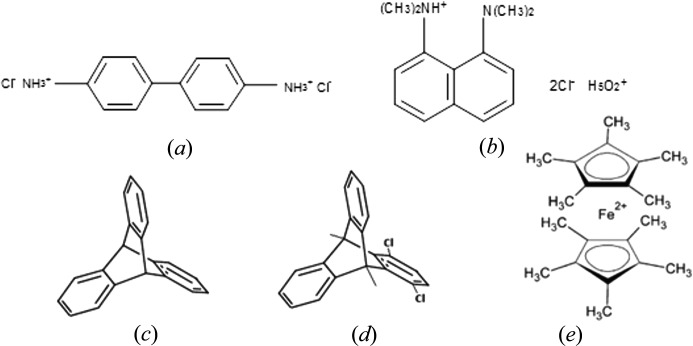
Definition of the molecules studied: (*a*) benzidine dihydrochloride (**BD^2+^ × 2Cl^−^**), (*b*) hydrated and protonated *N*,*N*,*N*,*N*-*peri*(dimethylamino)naphthalene chloride (**DMANH^+^ × 2Cl^−^ × H_5_O_2_^+^**), (*c*) triptycene (**T**), (*d*) dichlorodimethyltriptycene (**DCDMT**), (*e*) decamethylferrocene (Fc*).

For the purpose of our analysis we have chosen the following compounds: benzidine dihydrochloride (**BD^2+^ × 2Cl^−^**), hydrated and protonated *N*,*N*,*N*,*N*-*peri*(dimethylamino)naphthalene chloride (**DMANH^+^ × 2Cl^−^ × H_5_O_2_^+^**), triptycene (**T**), dichlorodimethyltriptycene (**DCDMT**) and decamethylferrocene (Fc*) as the model systems. Most of them were already studied and their structural details have already been published elsewhere (Hazell *et al.*, 1971 ▸; Dobrzycki & Woźniak, 2006 ▸; Hoser *et al.*, 2010 ▸; Makal *et al.*, 2010 ▸) or deposited in the CSD (Refcodes: CCDC 999149–999150, CCDC 999141–999142). However, the single-crystal neutron diffraction refinement results for decamethylferrocene, triptycene and dichlorodimethyltriptycene are new. All these data sets have variable quality and one of the purposes of this work is to see the influence of quality of X-ray diffraction data on results compared with the corresponding neutron results. Structural properties of **T** and **DCDMT** will soon be discussed in a separate paper (Hoser *et al.*, 2015 ▸). For all of them, we have both single-crystal neutron reference geometries and the results of X-ray diffraction data collection. All the molecules studied are illustrated in Fig. 1 ▸ and their most important parameters are given in Table 1 ▸.

For each compound, a series of IAM and, for **BD^2+^ × 2Cl^−^**, **DMANH^+^ × 2Cl^−^×H_5_O_2_^+^** and **T**, also TAAM refinements against X-ray data with different values of the limiting 2θ_max_ diffraction angle were conducted and their results were compared to the neutron structural results used as the reference ones. We will present a number of dependences of different parameters characterizing the quality of X-ray data sets and the average differences between particular neutron and structural parameters on the 2θ_max_ diffraction angle.

## 2. Methodology   

The average difference between particular neutron and X-ray structural parameters of a given type (*B*_θ_) is defined as
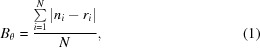
where *n_i_* is the *i*th value of the neutron structural parameter for a given studied molecule, *r_i_* is the *i*th value of the X-ray structural parameter, and *N* is the total number of parameters of a given type (for example, bond lengths between the non-H atoms, valence angles for heavy atoms, or similar parameters defined including H atoms).

First of all, all structural refinements of X-ray data were carried out in *SHELX* (Sheldrick, 2008 ▸) by modifying the refinement instruction input files (*.ins) by adding a proper OMIT instruction. The OMIT instruction allows definition of a limiting 2θ_max_ angle above which reflections are ignored. The value of the θ_max_ diffraction angle was increased by 1° in the range of θ_max_ angles from 20 to 40°. Then the refinement process based on |*F*|^2^ was carried out using *SHELX* for each of these diffraction θ_max_ angle values. Finally, the *B*_θ_ values were calculated and plotted. As already mentioned in §1 all X-ray data sets have different quality including intentionally incomplete data for **DCDMT** (see Table 1 ▸). The Friedel pairs in the *hkl* data sets were not merged for **DCDMT** as there are Cl atoms in this structure, but were merged for **T** as in this case the molecule consists of only H and C atoms and the anomalous differences are not significant. The results obtained are presented as a series of diagrams showing a number of dependences of different parameters characterizing the quality of X-ray data sets and the average differences between particular neutron and X-ray structural parameters as a function of the 2θ_max_ diffraction angle for a series of the studied model molecules.

### 2.1. Neutron measurements   

Neutron experiments were performed on the time-of-flight (TOF) single-crystal Laue diffractometer (SXD) (Keen *et al.*, 2006 ▸) at ISIS (Oxfordshire, UK). The data were collected at 100 K. The integration process was carried out with *SXD*2001 (Guttmann, 2005 ▸). The structures were refined using *SHELXL* (Sheldrick, 2008 ▸) with the single batch of wavelength and extinction-corrected reflections. Crystallographic data are given in the supporting information. In the case of the neutron data, we have checked the Hirshfeld rigid-bond test (see the supporting information) which supplies excellent results showing that the rigid-bond approximation is well fulfilled (practically almost no DMSDA values larger than 0.0010 Å^2^).

### 2.2. TAAM refinements   

For **BD^2+^ × 2Cl^−^**, **DMANH^+^ × 2Cl^−^ × H_5_O_2_^+^** and **T**, a series of TAAM refinements have been performed varying the resolution range. Initial atomic coordinates and ADPs for each compound were taken from the IAM refinements. Initial multipolar parameters and contraction–expansion parameters were transferred from the University of Buffalo Data Bank (UBDB) with the aid of the *LSDB* program (Jarzembska & Dominiak, 2012 ▸). The multipole expansion was truncated at the hexadecapole (*l*_max_ = 4) level for the non-H atoms, whereas at the quadrupole (*l*_max_ = 2) level for H atoms. TAAM refinements based on |*F*|^2^ have been performed in the *MOPRO* package (Guillot *et al.*, 2001 ▸). Statistical weights were used. The refinement strategy was as follows: (1) scale factor; (2) scale factor, atomic coordinates and ADPs for the non-H atoms; (3) scale factor, atomic coordinates and ADPs for the hydrogen atoms; (4) scale factor, atomic coordinates and ADPs for all atoms. *MOPRO* allows the resolution range for the refinement procedure to be specified. For our purpose, all refinements for a given structure started from the same initial model and were performed utilizing different specified resolution ranges.

### 2.3. ADP analysis   

In order to investigate differences in ADPs obtained after IAM and TAAM refinements against different resolution data, we decided to employ the similarity index. This index was introduced by Whitten & Spackmann (2006 ▸) and it is defined as
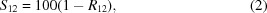
where 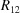 is a measure of the overlap between the probability density functions described by two ADPs **U1** and **U2** (in the Cartesian frame)
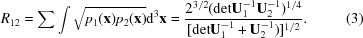
For two identical ADPs 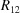 = 1.0, and 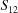 = 0. The smaller value of 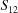 the better agreement between **U1** and **U2**.

### 2.4. Crystal entropy evaluation   

It has been shown by Bürgi and co-workers (Aree & Bürgi, 2006 ▸, 2012 ▸; Bürgi & Capelli, 2000 ▸; Capelli *et al.*, 2000 ▸) that analysis of multi-temperature X-ray or neutron diffraction data may afford thermodynamic information, *e.g.* specific heats, when a rigid-body or semi-rigid body approach is applied to the ADPs. Subsequent results reported by Madsen & Larsen (2007 ▸; Madsen *et al.*, 2011 ▸) indicate that in the case of low-temperature structures a single-temperature high-resolution X-ray measurement may be sufficient, provided that good quality ADPs are obtained. Having accurate estimates of ADPs, it is possible to calculate vibrational entropies *S_TLS_* corresponding to the low-frequency lattice vibrations. To derive the vibrational entropy associated with the low-frequency modes, one should conduct the TLS analysis (Schomaker & Trueblood, 1968 ▸, 1998 ▸; Cruickshank, 1956 ▸; Sands, 1982 ▸) and compute the related frequencies (and some estimated standard uncertainties, based on the standard uncertainties given for the eigenvalues of the TLS-fit matrix in the *THMA* program; Schomaker & Trueblood, 1968 ▸). *R*_TLS_, which help to judge the quality of the TLS-fit and indicates how much ADPs calculated after TLS analysis differ from those obtained after the X-ray diffraction experiment, is defined as: 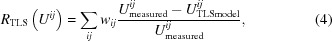
where *w_ij_* is the weight used in the least-squares fit of the TLS model against the observed ADPs 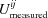. After TLS analysis, the vibrational entropy of crystals as a function of temperature can then be calculated as the sum of the contributions from each oscillator
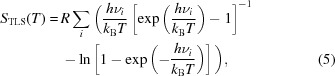
where *R* is the gas constant, *k*_B_ is Boltzmann’s constant, and *v_i_* is a frequency of a given *i*th oscillator. Following the above procedure, the vibrational entropy related to the low-frequency modes was estimated on the basis of the collected X-ray diffraction data with different resolution cut-offs and approaches of obtaining ADPs.

## 3. Results and discussion   

### 3.1. (Reflections/parameters) ratio *versus* 2θ_max_ diffraction angle   

The ratio of the number of independent reflections to the number of calculated parameters of a model fitted during least-squares refinement informs us about the feasibility and reliability of the refinement. Strict statisticians expect this ratio to be above 10, whereas more liberal statisticians expect it to be at least 5 in order to obtain meaningful results of the refinement. Of course, this ratio strongly depends on the 2θ_max_ diffraction angle. This parameter is one of a few which contribute to the quality of diffraction data. However, there are a few other parameters which are equally – or even more – important than this ratio, for example: averaged *I*/σ(*I*), *R*_int_ or *R*_sigma_. Figs. 6S and 7S in the supporting information illustrate dependences of the *R*_merged_ value on the diffraction 2θ_max_ angle and I/σ *versus* resolution of data. In fact, only the analysis of all such parameters can give a reliable estimation of the quality of measured X-ray, or neutron, diffraction data.

In general, the reflection-to-parameters ratio increases as the 2θ_max_ angle increases and, for data with 100% completeness, it is *ca* 10 reflections per parameter for 2θ_max_ equal to *ca* 50° for small organic molecules with no heavy atoms. We present the dependence of the (reflections/parameters) ratio on the 2θ_max_ diffraction angle for our five studied molecules in the supporting information (Fig. 1S). One may say that these are typical data sets collected with 4-axis X-ray diffractometers using Mo *K*α radiation. In general, we wanted to have X-ray data sets of variable quality and one of them (**DCDMT**) intentionally has poor completeness over the whole resolution range.

### 3.2. *R*_all_ *versus* 2θ_max_ diffraction angle   

**Figure 2:**
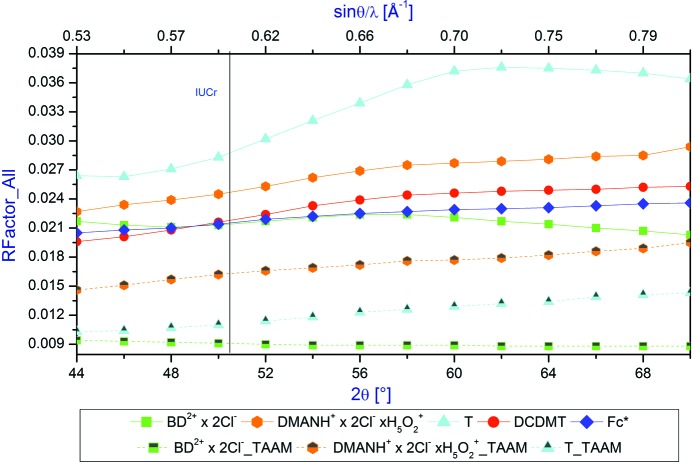
Dependence of the *R*_all_ factor on the 2θ_max_ diffraction angle in the range of angles 44–70°.

An agreement between the measured diffraction data and the refined model is characterized by discrepancy factors, for example, the residual factor for all reflections included in the refinement (*R*_all_) defined as
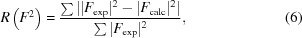
where *F* is the structure factor, and |*F*|^2^ represents the intensity of reflections. All refinements applying the IAM and TAAM models were carried out on the *F* ^2^ values. The dependence of the discrepancy factor *R*_all_ on the diffraction 2θ_max_ angle in the range of angles from 44 to 70° is illustrated in Fig. 2 ▸.

Firstly, one can see really significant differences in the values of *R*_all_ resulting from the use of IAM and TAAM models. Within the whole range of resolutions, the TAAM *R*_all_ values are in the range 1–2%, which are *ca* 2–3 times smaller than the corresponding IAM *R*_all_ parameters. This is a clear demonstration of the superiority of the TAAM model over IAM. However, similarly as in the case of the refinement of the IAM models, *R*_all_ slightly increases or decreases depending of the information content of the higher-resolution reflections.

As could be expected, because of the poor quality of the collected data, **T** exhibits the largest *R*_all_ discrepancy factor values. It is interesting that for the other low quality data set, the discrepancy factors are increasing with increasing values of the diffraction 2θ_max_ angle (see **DCDMT**). This means that the difference between the model refined and measured information resulting from experiment increases despite the increase in the number of measured reflections which are used in the refinement. In this case, more noise than information is added with the increasing 2θ_max_ diffraction angle to the *hkl* file. The above effect can also be attributed to the lack of description of the bonding density.

On the other hand, for two data sets, when the reflection-to-parameters ratio increases, the *R*_all_ factor value reaches a maximum (*ca* 56° for **BD^2+^ × 2Cl^−^** and 62° for **T**) and after that the discrepancy factors are decreasing as a function of diffraction angle. This means that together with the higher diffraction angle data more information than noise is collected. Similar relations are observed for *R*_GT_ and *wR*_GT_ (see Figs. 2S–4S in the supporting information). It is worth stressing that the IUCr limit (2θ_max_ = 50° for Mo *K*α) does not lead to any special values of the discrepancy factors.

### 3.3. Dependences of different geometrical parameters on 2θ_max_ diffraction angle   

#### 3.3.1. Bond lengths between the non-H atoms *versus* 2θ_max_ diffraction angle   

**Figure 3:**
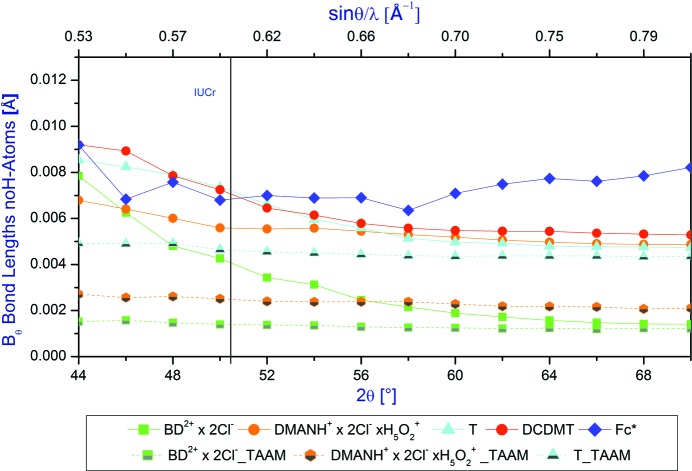
Average differences between the neutron and X-ray bond lengths for the non-H atoms as a function of the diffraction 2θ_max_ angle in the range of angles 44–70°.

The average differences between the neutron and X-ray bond lengths for the non-H atoms as a function of the 2θ_max_ diffraction angle are illustrated in Fig. 3 ▸.

In the case of the IAM refinements for all the model compounds analysed in this work, the average differences between the X-ray and neutron parameters decrease as a function of the increasing 2θ_max_ diffraction angle, with the exception of Fc*. The decrease is the most noticeable for the **BD^2+^ × 2Cl^−^** data. Most of the differences occur when 2θ_max_ is less than *ca* 56°. The average difference between the neutron and X-ray bond lengths for the non-H atoms is not greater than 0.010 Å, even for the low-quality data sets with poor resolution below the IUCr diffraction angle. When using the full resolution of the data this difference can drop to *ca* 0.0025 Å – or even less (for **BD^2+^ × 2Cl^−^**). With a resolution cut-off between 50 and 60°, the differences between X-ray and neutron models continue to drop and at 2θ_max_ = 60° it reaches a value which is *ca* 50% lower than for 2θ_max_ = 50°. This means that in order to have geometry of molecules closer to the neutron geometry, one should collect data to the higher 2θ_max_ diffraction angles as is common for routine X-ray data collection (and is common for experimental X-ray charge density studies).

It appears that TAAM refinements significantly improve agreement between the neutron and X-ray bond lengths for the non-H atoms. This is the case even for the low-resolution data, and it appears that the use of the resolution limit recommended by the IUCr is sufficient when the TAAM model is applied. The agreement between all results of IAM and TAAM refinements increases with the increasing resolution of data with the exception of Fc*.

#### 3.3.2. Bond lengths to H atoms *versus* the 2θ_max_ angle   

**Figure 4:**
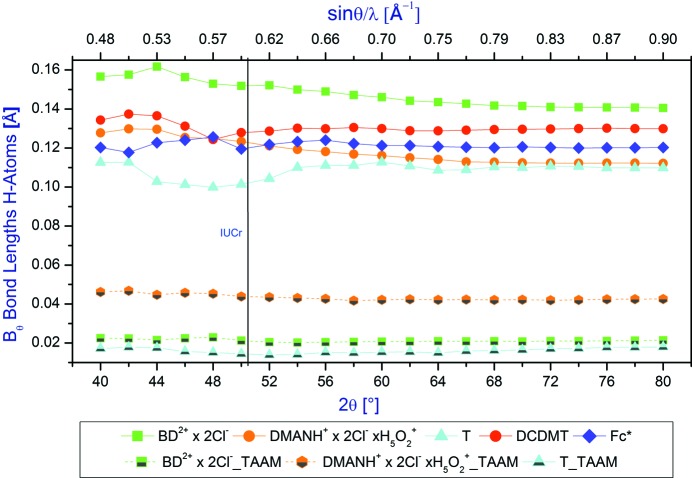
Average differences between the neutron and X-ray bond lengths to H atoms as a function of the 2θ_max_ diffraction angle.

The average differences between the neutron and X-ray bond lengths to H atoms as a function of the diffraction 2θ_max_ angle are illustrated in Fig. 4 ▸. When the TAAM model was applied no standardization of *X*—H bond lengths to the averaged neutron data were performed (although in general for other applications this is possible). The superiority of the TAAM model is obvious with the differences between the corresponding IAM and TAAM values are in the range *ca* 0.10–0.15 Å. Again, the TAAM average differences between the neutron and X-ray bond lengths to H atoms seem to be more or less the same within the whole range of resolutions, which means that even low-resolution data could be enough to obtain reliable geometry (when TAAM is applied).

When referring to the bond lengths of H atoms, the average differences between the neutron and X-ray data are one order of magnitude higher than those obtained for the non-H atoms. It is interesting to note that for the bond lengths to H atoms the differences between the X-ray and neutron values are quite different for all compounds (in the range *ca* 0.09 Å to almost 0.16 Å). All these differences are only slightly decreasing with the increase of the 2θ_max_ diffraction angle. As normally seen in structural analysis, the *X*—H bonds are constrained to the average neutron values for a given *X*—H bond type; the average corrections for particular compounds will be different as the compounds consist of a different number of *X*—H bonds of a particular type. A typical range of changes of the average differences between the neutron and X-ray bond lengths to H atoms with increasing 2θ_max_ diffraction angle is *ca* 15%.

#### 3.3.3. Valence angles for the non-H atoms *versus* 2θ_max_ diffraction angle   

**Figure 5:**
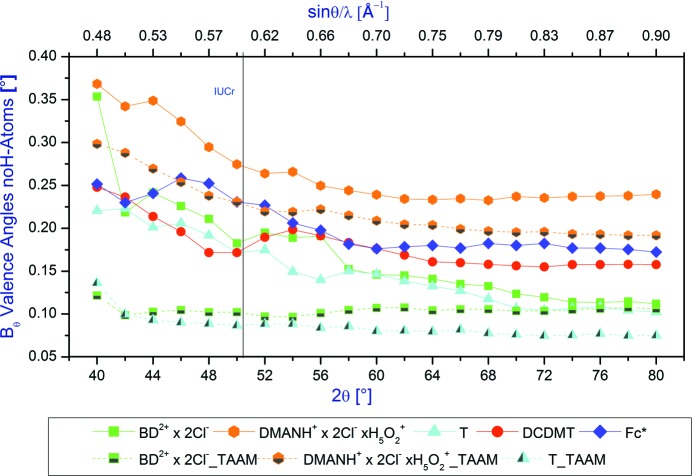
Average differences between the neutron and X-ray valence angles for the non H atom as a function of the diffraction 2θ_max_ angle in the range of angles 40–80°.

The average differences between the neutron and X-ray valence angles formed by the non-H atoms as a function of the 2θ_max_ diffraction angle in the range of angles from 40 to 80° are shown in Fig. 5 ▸.

We observe a similar pattern for the valence angles as was seen for the bond lengths between non-H atoms. The agreement between X-ray and neutron valence angles increase with the resolution. As in the case of the other previously analysed parameters, the IUCr limit does not seem to be particularly justified. However, when the higher-resolution X-ray data with reflections collected over a relatively large 2θ range are used, the average differences between the neutron and X-ray valence angles for the non-H atoms can be as small as *ca* 0.15–0.20°. Again the TAAM is superior to the IAM. The difference between the average X-ray and neutron valence angles for the non-H atoms obtained by applying IAM and TAAM decreases with increasing resolution. However, for the 2θ_max_ angle = 50° (Mo *K*α) such differences are significant with the valence angles obtained from TAAM being at least two times closer to the neutron values than the corresponding parameters obtained using the IAM with the exception of data for the **DMAN** salt for which this difference is smaller (*ca* 0.05°).

#### 3.3.4. Valence angles of H atoms *versus* 2θ_max_ diffraction angle   

**Figure 6:**
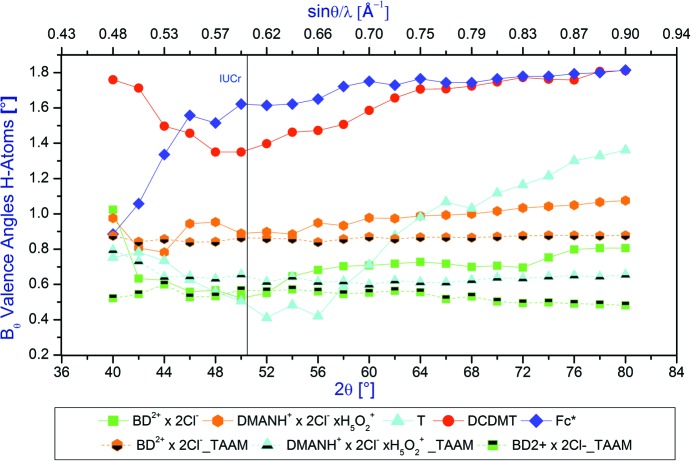
Average differences between the neutron and X-ray valence angles defined by H atoms as a function of the 2θ_max_ diffraction angle.

The average differences between the neutron and X-ray valence angles defined by H atoms as a function of the 2θ_max_ diffraction angle are shown in Fig. 6 ▸.

It appears that in the case of the valence angles defined by the inclusion of an H atom, the average difference between the neutron and X-ray values increases with increasing values of the 2θ_max_ diffraction angle. This is opposite to the relations obtained for the other structural parameters. The worse the quality of the *hkl* data set (Fc*, **T**, **DCDMT**), the larger differences between the average neutron and X-ray valence angles for H atoms (*ca* 1.7° for Fc* and **DCDMT**, *ca* 1.2 for **T** and *ca* 0.7° for **BD^2+^** × **2Cl^−^** and **DMANH^+^** × **2Cl^−^** × **H_5_O_2_^+^**). Similar to the other cases, the 2θ_max_ IUCr limit for the Mo *K*α radiation is doubtful. Above 2θ_max_ = 70°, the discrepancy between the average X-ray and neutron values becomes more or less constant with the exception of **T** for which it continuously increases in the whole range 56–80°. This means that the common assumption behind the standardization/normalization procedure of bond length to H atoms (*i.e.* that the valence angles to H atoms do not change when one lengthens the bond lengths to the average neutron values) is not fulfilled. This is one of the major reasons for problems with quantitative estimation of electron density distributions in charge density studies.

The average differences between the neutron and X-ray valence angles defined by H atoms as a function of the 2θ_max_ diffraction angle behave differently for IAM and TAAM. For TAAM they decrease with the increasing values of resolution, whereas the IAM differences increase. Again for smaller values of the TAAM average differences between the neutron and X-ray valence angles defined by H-atoms show the superiority of this model over the IAM.

#### 3.3.5. Comparison of ADPs from IAM and TAAM refinements   

**Figure 7:**
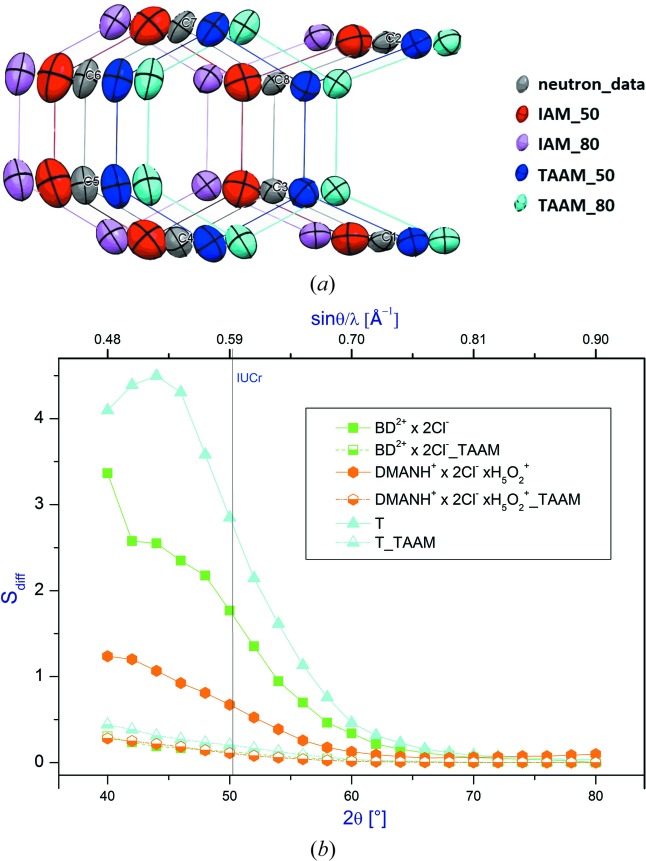
(*a*) Visualization of the ADP values obtained for the non-H atoms forming the independent part of the triptycene molecule. (*b*) The mean similarity index for ADPs obtained after IAM and TAAM compared with ADPs from TAAM refinement against high-resolution X-ray data as a function of the 2θ_max_ diffraction angle.

In Fig. 5S (supporting information), we plotted the overall mean similarity index (*S*_diff_) calculated when the non-H atom ADPs obtained for **BD^2+^ × 2Cl^−^**, **DMANH^+^ × 2Cl^−^ × H_5_O_2_^+^**, **T** after IAM and TAAM refinements against X-ray data cut to different resolutions are compared with the corresponding neutron values. It turns out that in the case of **T** and **BD^2+^ × 2Cl^−^**, the agreement between X-ray and neutron ADPs is significantly better when TAAM refinement is applied. This effect is less pronounced in the case of **DMANH^+^ × 2Cl^−^ × H_5_O_2_^+^**. In general, the values of the overall similarity index obtained for comparison of X-ray and neutron ADPs are high for the low-angle data. Fig. 7 ▸(*a*) presents visualization of the ADP values obtained for the non-H atoms forming the independent part of the triptycene molecule. There are significant differences between the ADPs calculated for data collected up to 2θ = 50° and those obtained for the larger diffraction angles (2θ = 80°). Of course the latter ones are closer to the neutron values of ADPs.

Similar dependences are obtained when one compares the non-H atom ADPs obtained from the IAM and from TAAM refinements against different resolution data to the ADPs obtained after TAAM refinement against the high-resolution X-ray data. Results are illustrated in Fig. 7 ▸(*b*). It appears that ADPs obtained after TAAM refinement conducted against low-resolution X-ray data are similar to those obtained after refinement of the high-resolution X-ray data. This analysis clearly demonstrates that ADPs obtained after the IAM refinement are inaccurate when only low-resolution data are used.

#### 3.3.6. TLS analysis and vibrational entropy estimation   

**Figure 8:**
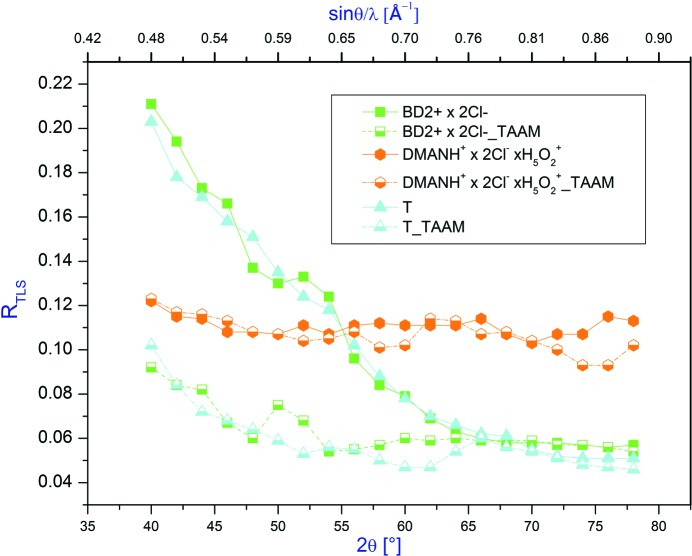
Dependences of the *R*_TLS_ values on resolution of X-ray data.

We have also investigated how resolution influences properties derived from ADPs, specifically the results of a TLS analysis. The systems used for the TLS analysis should be rigid. Thus, in the case of **BD^2+^ × 2Cl^−^** and **DMANH^+^ × 2Cl^−^ × H_5_O_2_^+^,** we used only the rigid cation molecules, *i.e.* the **DMANH^+^** and **BD^2+^** cations. It may be expected that these moieties for which the differences in ADPs obtained after TAAM and IAM refinements at low diffraction angles are large also exhibit significant differences in parameters obtained from the TLS analysis. First of all, *R*_TLS_ is significantly higher for triptycene and the benzidine cation after IAM refinement than after TAAM refinement below a resolution of 60° (see Fig. 8 ▸). The TLS analysis of well determined ADPs of truly rigid bodies (*e.g.* benzene) often gives *R*_TLS_(*U^ij^*) values of about 5%, especially for the low-temperature studies. For the less rigid systems values of 8–12% are common. We can immediately see that it is important to use data to at least the 2θ_max_ diffraction angle > 65° in order to make conclusions regarding the TLS analysis.

Additionally, for the **BD^2+^** cation and **T**, there are differences in frequencies obtained for normal modes related to translations and librations for IAM and TAAM refinements (see the supporting information). In the case of **T**, the rigid body refinement yields a non-physical result, and thus give no frequencies for resolution below 52°, and in the case of the benzidinium cation – below 48°. Application of TAAM gives a meaningful TLS refinement even at the low resolution.

In the case of the **DMANH^+^** cation, for which ADPs obtained at the low resolution only slightly differ from those obtained for the high-resolution data, *R_TLS_* and normal mode frequencies are also not changing either with resolution, or with different refinement methods applied.

**Figure 9:**
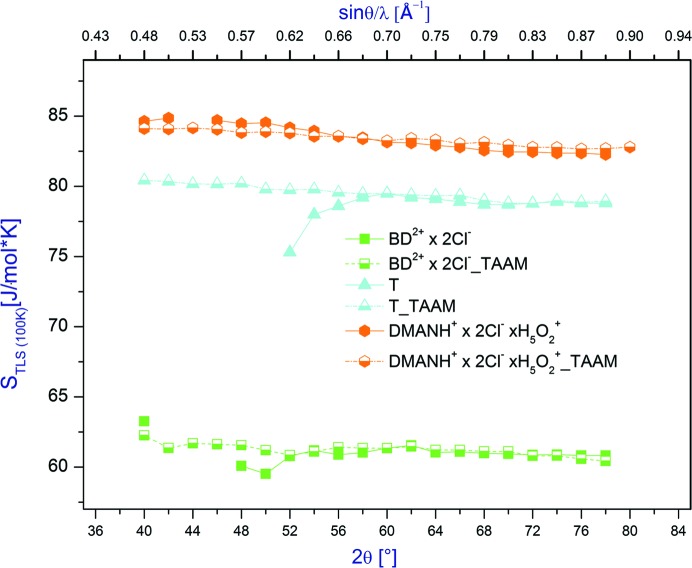
Vibrational entropy at 100 K *versus* resolution of X-ray data for **BD^2+^**, **DMANH^+^** and **T**.

The entropy estimation method proposed by Madsen & Larsen (2007 ▸) rely on frequencies obtained from TLS analysis. Thus, for triptycene and for the benzidine cation, for the IAM refinements against low-resolution data, when it is impossible to derive frequencies from TLS analysis, it is also impossible to estimate the part of vibrational entropy that is due to low-frequency phonons (Fig. 9 ▸). In such a case, the TAAM refinement, which enables estimation of normal mode frequencies even from low-resolution data, gives a unique opportunity to estimate vibrational entropy. However, it appears that entropies estimated at low-resolution data are higher by 2–3 J mol^−1^ K^−1^ (which at room temperature corresponds to *ca* 1 kJ mol^−1^) than those from the high-resolution data for all studied compounds and both models of electron density (see Tables 2S–4S and Figs. 8S and 9S in the supporting information and Fig. 9 ▸).

Even in the case of the **DMANH^+^** data which does not show significant differences between entropies obtained by using IAM and TAAM, the entropies obtained stabilize above 2θ_max_ > 65°. The same is true for the other compounds and for the difference in entropy values obtained at 298 and 100 K (Figs. 8S and 9S, and 9 ▸).

## 4. Conclusions   

A detailed analysis of the dependences of structural and thermal parameters obtained by X-ray diffraction on single crystals of five model compounds has been performed as a function of resolution of X-ray data. Two models – the Independent Atom Model and Transferable Aspherical Atom Model – were used in the refinement procedures. For all compounds – benzidine dihydrochloride, hydrated and protonated *N*,*N*,*N*,*N*-*peri*(dimethylamino)naphthalene chloride, triptycene, dichlorodimethyltriptycene and decamethylferrocene – the dependencies of the averaged differences between the X-ray and neutron corresponding geometrical parameters on the diffraction 2θ_max_ angle decrease with increasing resolution (with the exception of the valence angles defined by H atoms). The differences between the X-ray and neutron geometrical parameters can be significantly reduced when data are collected to the higher than commonly used 2θ_max_ diffraction angles (for example, for Mo *K*α 2θ_max_ > 65°). In the case of IAM models for the valence angles defined by H atoms, the smallest 2θ_max_ diffraction angles give the best agreement between the X-ray and neutron values, and discrepancy between the two increases with the increasing 2θ_max_ diffraction angle.

In order to obtain more adequate (more accurate and precise) structural and ADP parameters when the IAM model is used, one should collect data up to the larger diffraction angles, for Mo *K*α, at least, to 2θ_max_ = 65° (sin θ_max_/λ < 0.75 Å^−1^). Also the results of TLS analysis and vibrational entropy values are better for 2θ_max_ > 65°.

Stalke and co-workers (Krause *et al.*, 2015 ▸) suggested that it should be standard practice to collect data to the highest possible resolution when both heavy and light atoms are present. Our main conclusion is that even in the case of the light atoms only, the diffraction data should be collected to the highest resolution as this allows for refinement of more reliable structural, thermal and dependent parameters. Furthermore, the results of refinements using TAAM appear to be in better agreement with the neutron results than the corresponding IAM results for all parameters, all resolutions and all compounds, and for those who look for better quality structural parameters we advocate the use of this approach instead of the IAM.

## Supplementary Material

Crystal structure: contains datablock(s) BENZ_IAM_50, BENZ_IAM_80, BENZ_N_100, BENZ100D_overall, BENZ100D_phase_1, BENZ_TAAM_50, BENZ_TAAM_80, DCDMT_IAM_50, DCDMT_IAM_80, DCDMT_N_100, DMAN_IAM_50, DMAN_IAM_80, DMAN_N_100, DMAN_TAAM_50, DMAN_TAAM_80, FC_IAM_50, FC_IAM_80, FC_N_100, T_IAM_50, T_IAM_80, T_N_100, T_TAAM_50, T_TAAM_80. DOI: 10.1107/S2052252515020941/fc5011sup1.cif

Supporting tables and figures. DOI: 10.1107/S2052252515020941/fc5011sup2.pdf

Zipped archive of all CIF files. DOI: 10.1107/S2052252515020941/fc5011sup3.zip

CCDC references: 1442594, 1442595, 1442596, 1442597, 1442598, 1442599, 1442600, 1442601, 1442602, 1442603, 1442604, 1442605, 1442606, 1442607, 1442608, 1442609, 1442610, 1442611, 1442612, 1442613, 1442614

## Figures and Tables

**Table 1:** Symmetry and the unit-cell parameters for X-ray data collection of crystals of the studied compounds All experiments were conducted at 100 K.

	**BD^2+^ × 2Cl^−^**	**DMANH^+^ × 2Cl^−^ × H_5_O_2_^+^**	**T**	**DCDMT**	Fc*
System	Triclinic	Monoclinic	Orthorhombic	Monoclinic	Orthorhombic
Space group	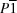	*P*2_1_/*n*	*P*2_1_2_1_2_1_	*P*2_1_	*Cmca*
Unit-cell dimensions					
*a* (Å)	6.571 (1)	10.085 (1)	8.0798 (3)	13.589 (3)	15.091 (1)
*b* (Å)	7.676 (1)	9.811 (1)	8.1645 (3)	8.042 (2)	11.4741 (8)
*c* (Å)	12.610 (2)	17.915 (1)	20.3778 (8)	14.943 (3)	9.9484 (6)
α (°)	85.17 (1)	90.0	90.0	90.0	90.0
β (°)	76.79 (1)	101.639 (1)	90.0	94.00 (2)	90
γ (°)	73.87 (2)	90.0	90.0	90.0	90.0
*R*_int_	0.031	0.029	0.030	0.032	0.023
*I*/σ	37.2	57.1	19.1	11.5	47.7
Completeness (%)	92	100	98.5	84	98
sin θ/λ (Å^−1^)	1.16	1.16	1.16	1.20	1.13
