# Supplementary material for: Yes, one can obtain better quality structures from routine X-ray data collection
Source: IUCrJ. 2016 Jan 1;3(Pt 1):61–70. doi: 10.1107/S2052252515020941 (PMC4704080; doi:10.1107/S2052252515020941)
Supplement: Supplementary file 2 [file m-03-00061-sup2.pdf]

IUCrJ

Volume 3 (2016)

Supporting information for article:

Yes, one can obtain better quality structures from routine X-ray data collection

W. Fabiola Sanjuan-Szklarz, Anna A. Hoser, Matthias Gutmann, Anders Østergaard Madsen and Krzysztof Woźniak

Supporting information contains:

- Table 1S. Neutron data collection parameters for **BD^{2+} × 2Cl^-**, **$DMANH^+$ × 2Cl^- × $H_5O_2^+$**, **T, DCDMT** and **Fc***.
- A series of figures from 1S to 9S defining respectively: dependence of the (number of reflection/number of parameters) ratio on the $2\theta_{max}$ diffraction angle, dependence of the R_{GT}-factor on $2\theta_{max}$ diffraction angle in the range of angles from 40º to 80º, dependence of the wR_{GT} factor on the $2\theta_{max}$ diffraction angle in the range of angles from 40º to 80º, dependence of the wR_{GT} factor on the diffraction $2\theta_{max}$ angle in the range of angles from 48º to 68º, dependence of the mean similarity index for ADPs (obtained after IAM and TAAM refinements) compared to ADPs from neutron data refinements on the diffraction $2\theta_{max}$ angle, dependence of the Rmerged and I/σ values on the diffraction $2\theta_{max}$ angle, I/σ versus resolution of data, vibrational entropy at room temperature versus resolution of X-ray data for **BD^{2+}**, **$DMANH^+$** and **T,** differences in vibrational entropy at room temperature and 100 K versus resolution of X-ray data for **BD^{2+}**, **$DMANH^+$** and **T.**
- Table 2S. Results of TLS analysis for BD^{2+}cation.
- Table 3S. Results of the TLS analysis for $DMANH^+$ cation.
- Table 4S. Results of the TLS analysis for triptycene.
- Results of the Hirshfeld rigid bond test for neutron data.

Table S1 Neutron data collection parameters and crystal data.

	**BD^{2+}×2Cl^-**	**DMANH$^+$×2Cl^- ×$H_5O_2^+$**	**T**	**DCDMT**	**Fc***
**System**	Triclinic	Monoclinic	Orthorhombic	Monoclinic	Orthorhombic
**Space group**	P-1	$P2_1/n$	P 2_1 2_1 2_1	$P2_1$	Cmca
**Unit cell dimensions**					
a/ Å	6.5742(10)	10.0638(3)	8.1019(13)	13.589(3)	15.119(4)
b/ Å	7.6702(12)	9.7890(3)	8.1922(13)	8.0415(16)	11.492(3)
c/ Å	12.6360(19)	17.8746(6)	20.442(3)	14.943(3)	9.967(3)
α/°	85.260(11)	90.0	90.0	90.0	90.0
β/°	76.735(11)	101.583(2)	90.0	93.998(13)	90.0
γ/°	73.823(11)	90.0	90.0	90.0	90.0
V($Å^3$)	595.50(16)	1725.05(9)	1356.8(4)	1628.9(5)	1731.7(8)
F(000)	131	211	322	422	121
Data, restrains, parameters	4109, 0, 271	3189,0,397	27163, 0, 308	12392, 1, 722	3378, 0, 125
GooF	1.138	1.388	1.075	1.560	1.88
R indices, all data	R=0.0770, wR=0.1675	R=0.0741, wR=0.2417	R=0.0726, wR=0.1616	R=0.0724, wR=0.1923	R=0.0886, wR=0.2442

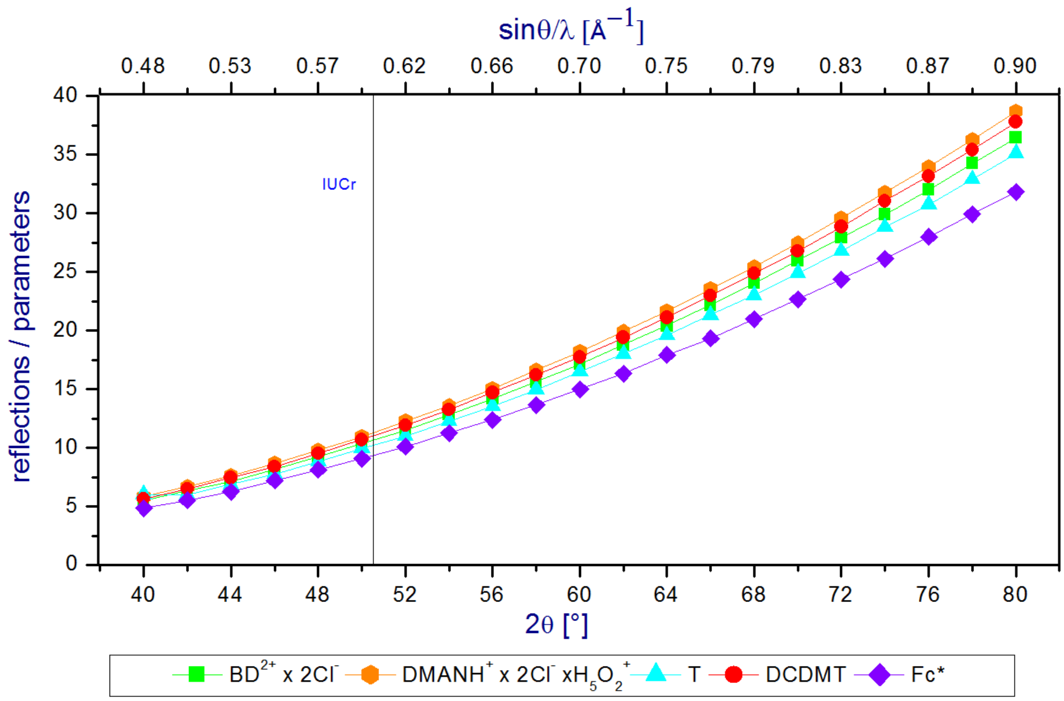

Figure S1 Dependence of the (number of reflection/number of parameters) ratio on the diffraction $2\theta_{max}$ angle.

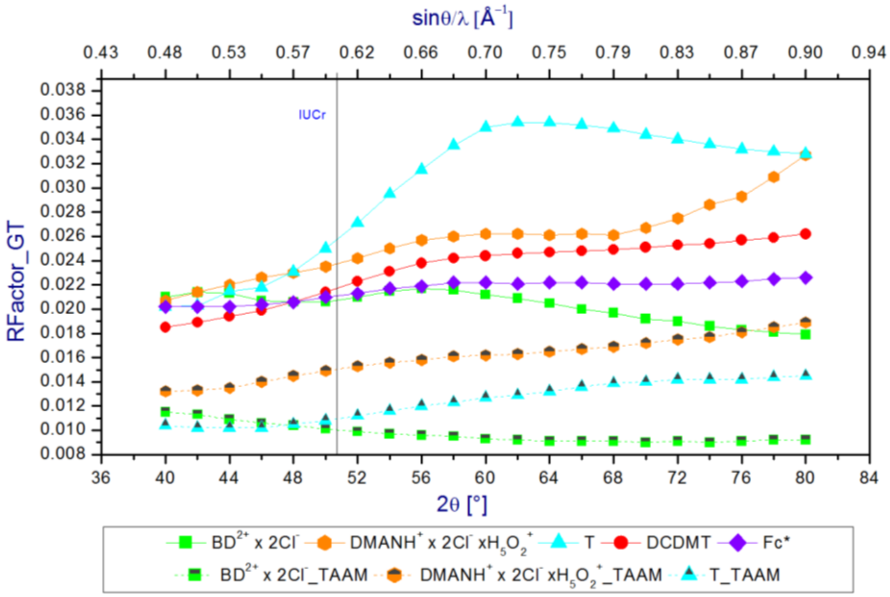

Figure S2 Dependence of the R_{GT}-factor on the $2\theta_{max}$ diffraction angle in the range from 40° to 80°.

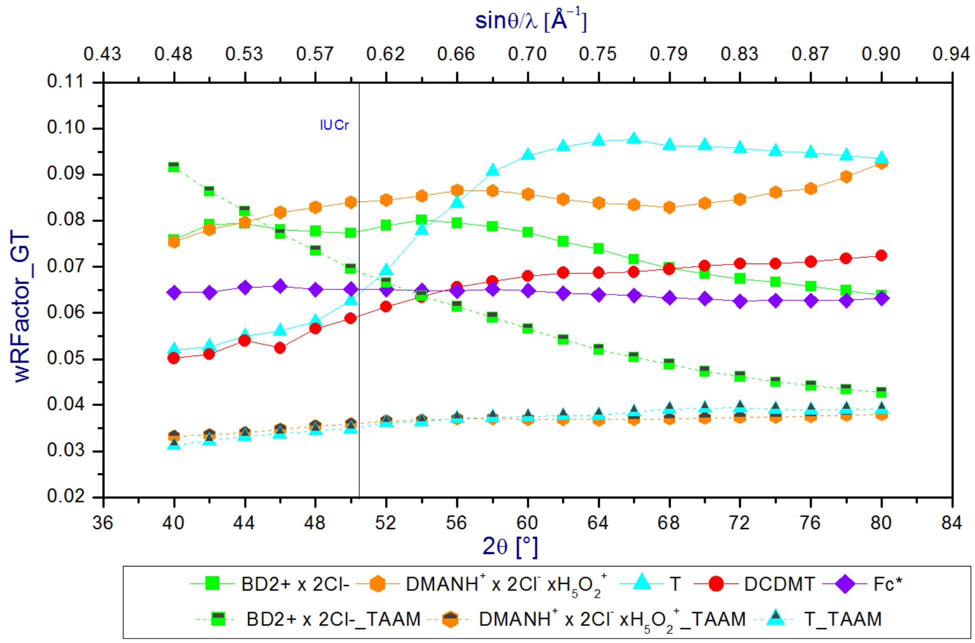

Figure S3 Dependence of the wR_{GT}-factor on the $2\theta_{max}$ diffraction angle in the range from 40º to 80º.

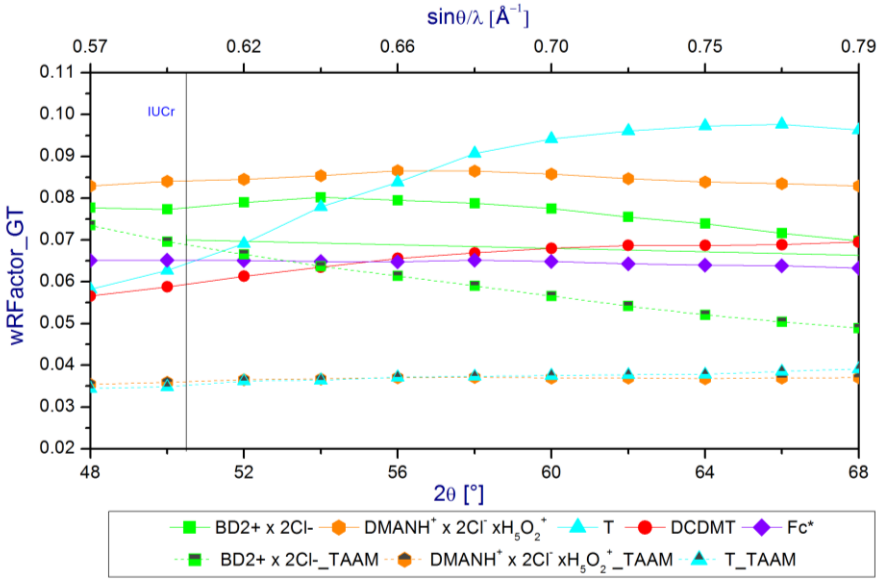

Figure S4 Dependence of the wR_{GT}-factor on the $2\theta_{max}$ diffraction angle in the range from 48º to 68º.

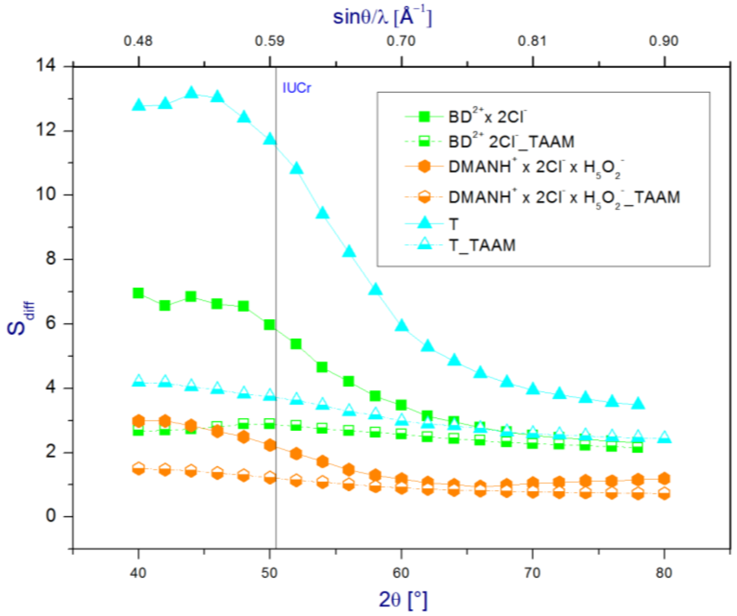

Figure S5 Dependence of the mean similarity index for ADPs (obtained after IAM and TAAM refinements) compared to ADPs from neutron data refinements, on the diffraction $2\theta_{max}$ angle.

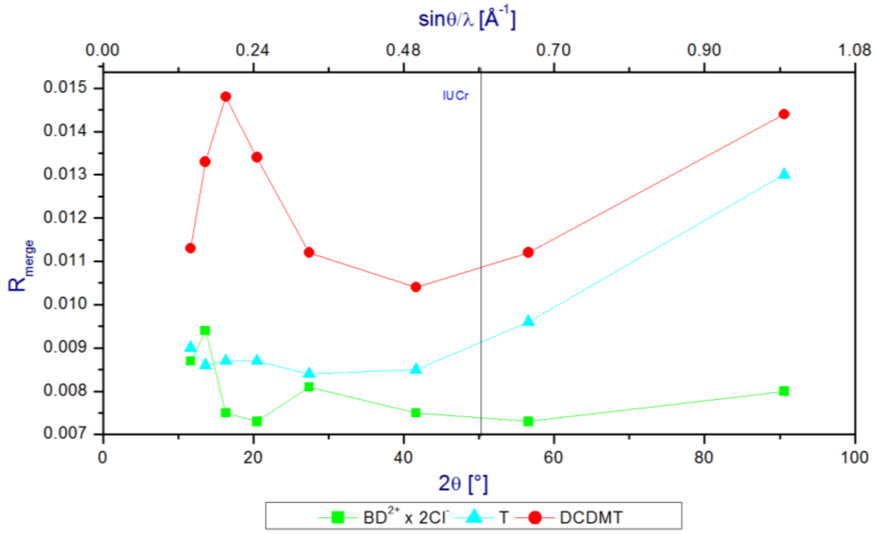

Figure S6 Dependence of the Rmerged value on the diffraction $2\theta_{max}$ angle.

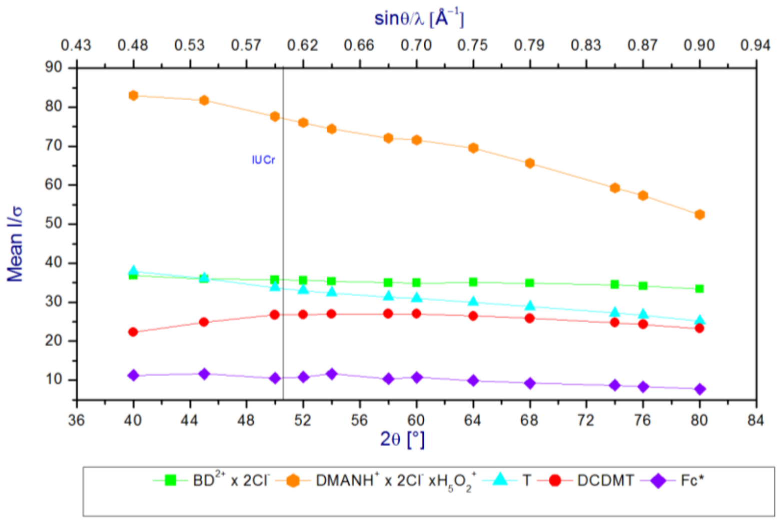

Figure S7 I/σ vs resolution of data.

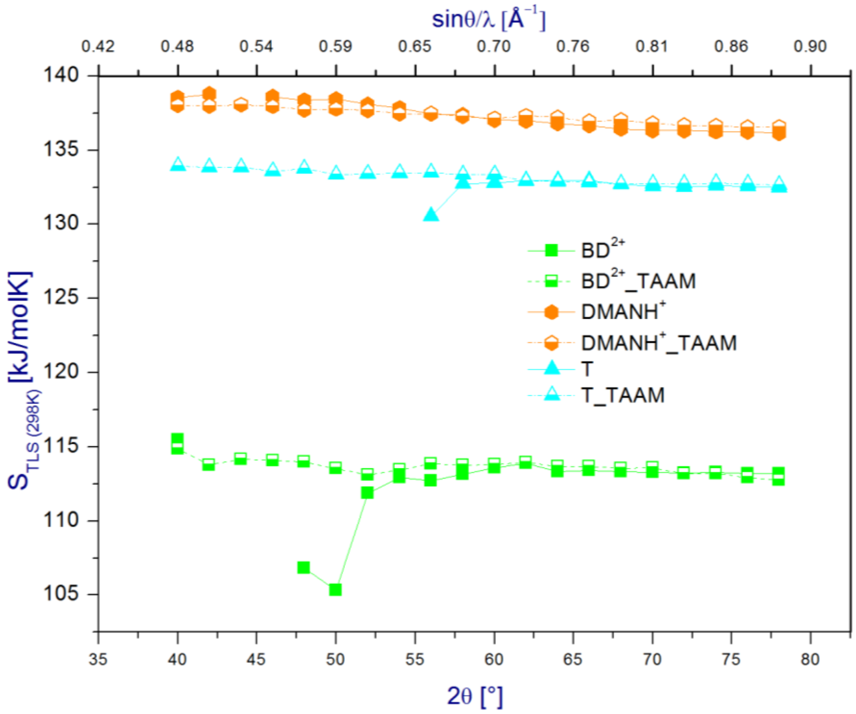

Figure S8 Vibrational entropy at room temperature versus resolution of X-ray data for BD^{2+}, $DMANH^+$ and T.

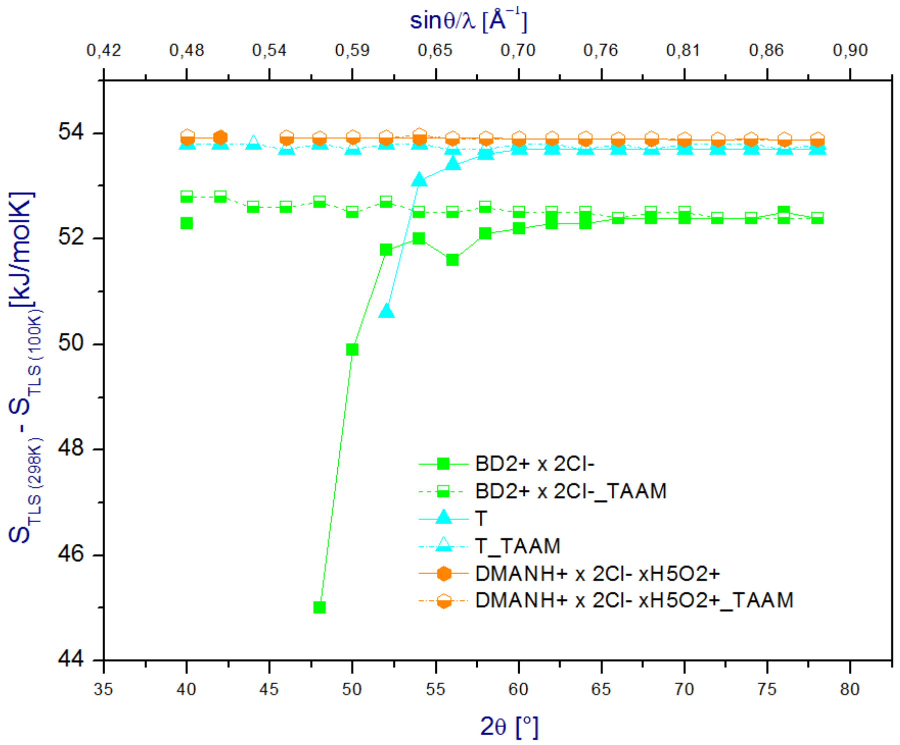

Figure S9 Differences in vibrational entropy at room temperature and 100K versus resolution of X-ray data for BD^{2+}, $DMANH^{+}$ and T.

Table S2 Results of TLS analysis for the benzidine cation.

IAM

$2\theta_{max}$	R_{TLS}	*Normal Mode Frequencies [cm-1]*						S_{TLS}^{100K}	S_{TLS}^{298K}
**40**	0.215	31.6	31.8	49.7	56.3	66.4	164.1	63.26	115.49
**42**	0.194	30.8	32.1	41.1	67.8	72.4	-	-	-
**44**	0.176	30.3	31.3	39.9	69.8	77.3	-	-	-
**46**	0.164	-						-	-
**48**	0.149	30.6	31.6	38.7	71.2	74.1	550.8	60.08	106.85
**50**	0.135	31.1	32.1	37.4	72	79	685.6	59.51	105.33
**52**	0.131	31.8	33.2	36.6	68.5	82.5	221.8	60.77	111.88
**54**	0.121	32.4	34.3	35.9	68.6	84.4	181.9	61.19	112.94
**56**	0.103	32.9	35.2	35.5	69	87.1	174.2	60.88	112.73
**58**	0.093	33.3	35.2	36	69.7	89.2	155.3	61.03	113.14
**60**	0.087	33.5	35.4	36.6	66.9	91	145.2	61.35	113.61
**62**	0.07	33.9	35.8	37.3	65	90.9	138.2	61.54	113.91
**64**	0.061	33.8	35.9	37.6	65.2	93.8	142.6	61.04	113.31
**66**	0.061	34	36.2	38	64.5	95	136.6	61.08	113.43
**68**	0.06	34	36.4	38.3	64.5	96	134.8	60.98	113.34
**70**	0.059	34	36.5	38.5	64.2	96.5	134.5	60.93	113.29
**72**	0.059	34	36.6	38.8	64.2	97.8	132	60.87	113.24
**74**	0.058	34	36.7	38.9	63.9	98.4	130.4	60.89	113.29
**76**	0.057	34	36.7	39	63.9	99.7	129.6	60.82	113.21
**78**	0.057	34	36.8	39.1	63.7	99.5	129.5	60.82	113.21

TAAM

$2\theta_{max}$	R_{TLS}	*Normal Mode Frequencies [cm-1]*						S_{TLS}^{100K}	S_{TLS}^{298K}
**40**	0.092	35.3	35.3	39.9	54.6	92.5	124.2	62.26	114.86
**42**	0.084	34.7	34.7	39	57.3	98.4	132	61.35	113.77
**44**	0.082	34.6	34.6	38.7	57.7	97.5	128	61.69	114.17
**46**	0.067	34.3	34.3	38.3	59.3	98.7	126.9	61.62	114.09
**48**	0.06	34.1	34.1	37.9	59.8	98.9	130.3	61.55	113.97
**50**	0.075	33.9	33.9	37.6	59.4	104.9	132.1	61.19	113.53
**52**	0.068	33.7	33.7	37.9	59.4	108.3	134.1	60.84	113.11
**54**	0.054	33.5	33.5	38	60.8	102.9	132.5	61.09	113.44
**56**	0.055	33.5	33.5	38.4	60.8	102.3	125.5	61.41	113.86
**58**	0.057	33.4	33.4	38.6	60.9	103.6	124.6	61.34	113.78
**60**	0.06	33.5	33.5	38.7	60.9	105.6	121.4	61.33	113.79
**62**	0.059	33.6	33.6	39	60.4	104.2	119.9	61.46	113.95

**64**	0.06	33.6	33.6	39.1	60.6	104	123.4	61.22	113.67
**66**	0.059	33.6	33.6	39.3	60.8	103.9	122	61.21	113.68
**68**	0.059	33.6	33.6	39.3	61.1	106	120.6	61.11	113.57
**70**	0.059	33.6	33.6	39.6	60.9	104.4	121.1	61.11	113.58
**72**	0.057	33.5	33.5	39.7	61.6	106.4	123.4	60.79	113.20
**74**	0.057	33.6	33.6	39.5	61.8	106	123.9	60.80	113.21
**76**	0.056	33.6	33.6	39.7	61.6	106	127.7	60.57	112.93
**78**	0.054	33.6	33.6	39.8	61.7	106.6	128.7	60.43	112.76

Table S3 Results of TLS analysis for triptycene.

IAM

**2θ$_{max}$**	**R$_{TLS}$**	***Normal Mode Frequencies [cm-1]***						***S$_{TLS}$^{100K}***	***S$_{TLS}$^{298K}***
**40**	0.159	-						-	-
**42**	0.158	-						-	-
**44**	0.145	-						-	-
**46**	0.14	-						-	-
**48**	0.141	-						-	-
**50**	0.132	-						-	-
**52**	0.13	-	-	-	-	-	-	-	-
**54**	0.127	23.6	24.7	26.3	41.8	52.8	-	-	-
**56**	0.112	24	25.6	27.4	41.1	52.4	136.7	77.54	130.53
**58**	0.097	24.8	26.4	28.3	41.6	55.2	88.5	79.17	132.73
**60**	0.08	25.2	27	29.3	41.9	56.1	79.5	79.18	132.81
**62**	0.071	25.7	27.6	29.9	42.5	56.8	71.2	79.29	132.98
**64**	0.064	26	27.9	30	42.9	58.6	66.9	79.23	132.95
**66**	0.061	26.2	28.2	30.5	43.1	57.9	64.9	79.24	132.97
**68**	0.058	26.4	28.5	30.9	42.9	58.3	64.8	78.98	132.7
**70**	0.055	26.6	28.7	31.2	43.5	58.2	63.5	78.84	132.57
**72**	0.051	26.7	28.8	31.4	43.4	58.5	62.7	78.8	132.54
**74**	0.049	26.8	28.9	31.6	43.4	58	61.8	78.87	132.61
**76**	0.05	26.9	29	31.8	43.3	57.5	61.8	78.85	132.59

**78**	0.051	27	29.1	31.9	43.4	57.2	62.2	78.74	132.48

TAAM

**2θ_{max}**	**R_{TLS}**	***Normal Mode Frequencies [cm-1]***						***S_{TLS}^{100K}***	***S_{TLS}^{298K}***
**40**	0.087	26.2	27.4	30.3	44.5	54.3	61.7	80.17	133.94
**42**	0.075	26	27.4	30.2	44.2	55.5	62.3	80.07	133.83
**44**	0.067	26.1	27.4	30.3	43.7	54.8	63.2	80.09	133.85
**46**	0.061	26.1	27.5	30.3	43.7	56.7	63	79.81	133.57
**48**	0.064	26.2	27.8	30.5	43.8	55	62	79.98	133.76
**50**	0.06	26.2	27.8	30.6	44.4	55.6	63.2	79.61	133.37
**52**	0.051	26.2	27.9	30.9	44.1	55.3	62.9	79.64	133.4
**54**	0.05	26.3	28	31.1	44.2	54.9	61.9	79.69	133.46
**56**	0.049	26.5	28.4	31.4	43.7	54.5	60.9	79.71	133.48
**58**	0.058	26.7	28.6	31.4	43.3	55.2	60.7	79.59	133.36
**60**	0.057	26.8	28.9	31.8	43.9	54.7	58.9	79.56	133.35
**62**	0.055	26.8	29	31.9	44.3	54	61.9	79.15	132.92
**64**	0.049	26.9	29	32	44.6	53.9	61.3	79.13	132.9
**66**	0.049	27	29.1	32	44.3	53.7	61.8	79.09	132.86
**68**	0.049	27.1	29.3	32.2	44.5	53.5	61.7	78.96	132.73
**70**	0.048	27.2	29.3	32.3	44.5	53.5	61.1	78.98	132.75
**72**	0.047	27.2	29.4	32.3	44.4	53.2	61.5	78.97	132.73
**74**	0.048	27.3	29.4	32.4	44.1	53	61.3	79.02	132.79
**76**	0.048	27.3	29.5	32.4	44.1	53.2	61.2	78.98	132.75
**78**	0.049	27.3	29.5	32.5	44	53.2	61.8	78.89	132.66

Table S4 Results of TLS analysis for $DMANH^+$ cation.

**2θ_{max}**	**R_{TLS}**	***Normal Mode Frequencies [cm-1]***						***S_{TLS}^{100K}***	***S_{TLS}^{298K}***
**40**	0.122	23.5	24.6	31.9	39.1	49.4	52.2	84.64	138.55
**42**	0.115	23.6	24.3	32.2	38.9	47.8	52.7	84.86	138.78
**44**	0.114	-	-	-	-	-	-	-	-
**46**	0.108	23.8	24.5	32.4	37.6	48.4	53.7	84.70	138.61

IAM

**48**	0.108	23.9	24.7	32.5	37.6	48.4	54.4	84.47	138.38
**50**	0.107	24.3	25.1	32.6	37.9	47.3	52.8	84.53	138.45
**52**	0.111	24.6	25.5	32.5	38.2	47.7	52.9	84.18	138.09
**54**	0.107	24.9	25.8	32.8	37.8	48.3	52.6	83.94	137.85
**56**	0.111	25.3	26.4	32.9	37.8	48.1	53	83.56	137.47
**58**	0.112	25.7	26.8	33	36.6	48.4	53.2	83.47	137.38
**60**	0.111	25.7	27	33.1	36.9	48.9	53.8	83.15	137.04
**62**	0.111	25.9	27.3	33.4	37.1	48.5	52.8	83.09	136.99
**64**	0.111	26	27.6	33.2	36.6	48.7	54	82.92	136.81
**66**	0.114	26	27.8	32.9	36.8	49.2	54.1	82.79	136.68
**68**	0.107	26.1	27.9	33.2	36.7	49.4	54.6	82.57	136.46
**70**	0.103	26.2	28.1	33.3	36.4	49.5	55	82.45	136.33
**72**	0.107	26.3	28.1	33.4	36.5	49.4	54.6	82.45	136.33
**74**	0.107	26.4	28.3	33.3	36.4	49.5	54.7	82.37	136.26
**76**	0.115	26.3	28.3	33.4	36.5	49.7	54.4	82.37	136.25
**78**	0.113	26.4	28.4	33.3	36.4	49.7	55	82.27	136.15

**2θ$_{max}$**	**R$_{TLS}$**	***Normal Mode Frequencies [cm-1]***						***S_{TLS}^{100K}***	***S_{TLS}^{298K}***
**40**	0.123	25.0	26.7	31.9	36.2	48.4	53.1	84.1137	138.0294
**42**	0.117	25.0	26.7	31.9	35.9	48.1	54.1	84.08337	137.9956
**44**	0.116	24.9	26.7	31.6	35.4	48.3	54.9	84.15851	138.068
**46**	0.113	25.0	26.9	31.8	35.5	48.5	54.2	84.05813	137.9688
**48**	0.108	24.9	26.8	31.7	35.3	48.5	56.8	83.82387	137.7188
**50**	0.107	25.1	27.0	31.7	35.3	48.8	55.1	83.88722	137.7907
**52**	0.104	25.2	27.2	32.0	35.5	48.6	54.5	83.79025	137.6959

TAAM

**54**	0.105	25.4	27.3	31.9	35.3	49.0	55.5	83.55772	137.4545
**56**	0.108	25.5	27.4	32.0	35.3	48.6	55.2	83.578	137.4782
**58**	0.101	25.6	27.6	32.1	35.4	48.6	55.6	83.38068	137.2762
**60**	0.102	25.8	27.8	31.9	35.2	50.3	54.4	83.25277	137.1464
**62**	0.114	25.5	27.6	32.4	35.3	50.0	53.6	83.42284	137.3228
**64**	0.113	25.7	28.0	32.2	35.4	49.4	53.9	83.32016	137.2202
**66**	0.107	25.8	28.3	31.9	35.4	49.4	55.5	83.04625	136.9353
**68**	0.108	26.0	28.2	32.1	35.1	48.9	55.3	83.13964	137.0333
**70**	0.104	26.0	28.3	32.1	35.1	49.4	55.9	82.94438	136.8306
**72**	0.100	26.4	28.6	31.7	35.4	49.5	55.6	82.79195	136.6775
**74**	0.093	26.4	28.6	31.8	35.5	49.0	56.0	82.76773	136.6527
**76**	0.093	26.1	28.4	32.3	35.2	50.1	56.1	82.669	136.5483
**78**	0.102	26.0	28.3	32.5	35.4	50.4	55.3	82.699	136.5806
**80**	0.104	26.1	28.4	32.1	35.5	50.0	55.2	82.7948	136.6797

S1. Results of the Hirshfeld rigid bond test

$BE^{2+}x2Cl^{-}$

Rigid-Body Model Libration Corrections for Bond Distances and "Hirshfeld Rigid-Bond" Test (Acta Cryst., 1976, A32, 239-244)

Bond		Bond Distance		Components of the Correction			MSDA from U(obs) Vibration Along the Interatomic Bond				Angle with Lib. Axes		
Atom(I)	Atom(J)	Obsd	Calcd	Del(L)	Del(M)	Del(N)	I to J	J to I	Difference	Sqrt(Diff)	L(1)	L(2)	L(3)
N(1)	- C(1)	1.4588(13)	1.4589	-0.0001	0	0	0.0062(3)	0.0051(4)	0.0011(5)	0.0332	179.69	90.08	90.10
N(2)	- C(7)	1.4641(13)	1.4643	0.0002	0	0.0001	0.0057(3)	0.0057(4)	0.0000(5)	0	2.42	89.04	87.77
C(1)	- C(2)	1.3890(15)	1.3907	-0.0001	-0.0019	-0.0004	0.0067(4)	0.0064(3)	0.0003(5)	0.0173	119.98	137.09	62.45
C(1)	- C(6)	1.3911(15)	1.3929	-0.0001	0.0020	0.0003	0.0065(4)	0.0066(3)	0.0001(5)	0.0100	118.68	43.52	119.60
C(2)	- C(3)	1.3957(14)	1.3958	-0.0001	0.0001	-0.0001	0.0061(3)	0.0052(3)	0.0009(5)	0.0300	177.80	90.27	92.18
C(3)	- C(4)	1.4059(14)	1.4076	-0.0001	0.0020	0.0003	0.0064(3)	0.0062(4)	0.0002(5)	0.0141	120.45	44.06	118.43
C(4)	- C(5)	1.4061(14)	1.4078	0.0001	0.0019	0.0004	0.0061(4)	0.0061(3)	0.0000(5)	0	58.33	43.58	116.54
C(4)	- C(10)	1.4853(13)	1.4854	-0.0002	0	-0.0001	0.0051(4)	0.0050(4)	0.0001(5)	0.0100	177.06	91.23	92.66
C(5)	- C(6)	1.3958(14)	1.3959	0.0001	0	0	0.0063(3)	0.0065(3)	0.0002(5)	0.0141	0.97	89.84	89.13
C(7)	- C(8)	1.3902(15)	1.3919	0	0.0019	-0.0003	0.0069(4)	0.0073(4)	0.0004(5)	0.0200	59.15	58.42	132.87
C(7)	- C(12)	1.3892(15)	1.3910	0.0001	-0.0020	0.0005	0.0068(4)	0.0071(4)	0.0003(5)	0.0173	62.23	119.12	42.36
C(8)	- C(9)	1.3921(14)	1.3922	0.0001	0	0.0002	0.0062(4)	0.0054(4)	0.0008(5)	0.0283	4.25	87.97	86.28
C(9)	- C(10)	1.4047(15)	1.4064	0.0001	-0.0020	0.0005	0.0064(4)	0.0065(4)	0.0001(5)	0.0100	60.46	118.29	43.15
C(10)	- C(11)	1.4036(14)	1.4052	0	-0.0019	0.0003	0.0059(4)	0.0063(4)	0.0004(5)	0.0200	122.58	121.10	48.27
C(11)	- C(12)	1.3941(14)	1.3943	-0.0001	0	-0.0002	0.0059(4)	0.0058(4)	0.0001(5)	0.0100	175.90	92.58	93.18

Sqrt(Sum(DelIJ**2)/Nrb) = 0.0005

# - Indicates bonds exceeding the 5.0 sigma test level

"BENZ100D_p" PLATON-ADP-Anal Page 18

Test Matrix for Rigid-Body Vibrations - /Del(A,B)/ = /Z(A,B)**2 - Z(B,A)**2/ Should be Near Zero (Acta Cryst. A34, 1978, 828)

Atom-Atom			1	2	3	4	5	6	7	8	9	10	11	12	13	14
1	N(1)	-	0	0	-1	0	1	1	0	0	0	0	1	1	0	0
2	N(2)	-	10	0	1	0	1	1	0	1	0	0	1	1	0	0
3	C(1)	-	-1	9	0	0	0	0	1	0	1	1	1	0	1	0
4	C(2)	-	2	8	-1	0	-1	1	0	0	0	0	0	1	0	0
5	C(3)	-	4	7	2	-1	0	0	1	0	0	1	0	0	1	0
6	C(4)	-	4	6	3	2	-1	0	0	0	1	1	0	0	0	0
7	C(5)	-	4	7	2	3	2	-1	0	0	0	0	1	1	0	1
8	C(6)	-	2	8	-1	2	3	2	-1	0	0	0	1	1	0	0
9	C(7)	-	9	-1	7	7	5	4	5	7	0	0	1	1	0	0
10	C(8)	-	8	2	7	6	5	4	4	6	-1	0	-1	0	1	0
11	C(9)	-	7	4	5	5	4	3	3	4	2	-1	0	0	0	0
12	C(10)	-	6	4	4	4	3	-1	3	4	3	2	-1	0	0	1
13	C(11)	-	7	4	5	4	3	3	4	5	2	3	2	-1	0	0
14	C(12)	-	8	2	7	6	4	4	5	6	-1	2	3	2	-1	0

Remarks

- Upper Triangle Entries Represent /Del(A,B)/*1000 Values

- Lower Triangle Entries Represent Distances (A-B) Angstrom

- Negative Entries Indicate Bonded Atoms

T - Triptycene

===

Rigid-Body Model Libration Corrections for Bond Distances and "Hirshfeld Rigid-Bond" Test (Acta Cryst., 1976, A32, 239-244)

===

Bond		Bond Distance		Components of the Correction			MSDA from U(obs) Vibration Along the Interatomic Bond				Angle with Lib. Axes		
Atom(I)	Atom(J)	Obsd	Calcd	Del(L)	Del(M)	Del(N)	I to J	J to I	Difference	Sqrt(Diff)	L(1)	L(2)	L(3)
C(1)	- C(3)	1.5319(12)	1.5329	0.0005	0.0008	-0.0005	0.0089(3)	0.0090(3)	0.0001(4)	0.0100	87.27	35.45	125.31
C(1)	- C(9)	1.5304(13)	1.5313	0.0003	-0.0007	-0.0005	0.0103(3)	0.0101(3)	0.0002(4)	0.0141	43.06	126.42	109.71
C(1)	- C(15)	1.5285(12)	1.5293	-0.0007	-0.0001	-0.0004	0.0102(3)	0.0105(3)	0.0003(4)	0.0173	149.00	116.98	104.10
C(2)	- C(8)	1.5291(12)	1.5301	0.0004	0.0008	0.0004	0.0102(3)	0.0104(3)	0.0002(4)	0.0141	84.08	12.34	79.21
C(2)	- C(14)	1.5290(10)	1.5299	0.0003	-0.0007	0.0004	0.0089(3)	0.0084(3)	0.0005(4)	0.0224	37.69	115.81	64.58
C(2)	- C(20)	1.5254(14)	1.5262	-0.0008	-0.0001	0.0005	0.0051(3)	0.0049(3)	0.0002(4)	0.0141	142.65	107.53	58.22
C(3)	- C(4)	1.3902(12)	1.3911	0.0004	0.0007	0.0005	0.0083(3)	0.0095(4)	0.0012(5)	0.0346	82.60	19.95	71.59
C(3)	- C(8)	1.4094(12)	1.4105	0	0	-0.0010	0.0086(3)	0.0075(3)	0.0011(4)	0.0332	94.69	101.61	167.46
C(4)	- C(5)	1.4034(14)	1.4044	0.0004	0.0007	-0.0005	0.0129(4)	0.0133(4)	0.0004(5)	0.0200	86.85	42.58	132.41
C(5)	- C(6)	1.3968(14)	1.3978	0	0	-0.0010	0.0193(4)	0.0204(4)	0.0011(5)	0.0332	94.31	101.55	167.64
C(6)	- C(7)	1.4040(12)	1.4049	-0.0004	-0.0007	-0.0005	0.0150(4)	0.0149(4)	0.0001(5)	0.0100	97.94	159.86	108.38
C(7)	- C(8)	1.3924(13)	1.3934	-0.0004	-0.0007	0.0005	0.0065(4)	0.0061(3)	0.0004(5)	0.0200	92.98	137.46	47.61
C(9)	- C(10)	1.3915(10)	1.3923	0.0003	-0.0006	0.0005	0.0083(3)	0.0087(3)	0.0004(4)	0.0200	40.17	111.33	57.81
C(9)	- C(14)	1.4085(12)	1.4096	0	0	-0.0010	0.0074(3)	0.0069(3)	0.0005(4)	0.0224	94.41	101.74	167.44
C(10)	- C(11)	1.4058(14)	1.4066	0.0003	-0.0006	-0.0005	0.0147(3)	0.0150(3)	0.0003(5)	0.0173	46.90	124.80	117.08
C(11)	- C(12)	1.3962(13)	1.3972	0	0	-0.0010	0.0162(3)	0.0165(4)	0.0003(5)	0.0173	94.40	101.97	167.22
C(12)	- C(13)	1.4053(11)	1.4061	-0.0003	0.0006	-0.0005	0.0114(4)	0.0113(4)	0.0001(5)	0.0100	139.79	68.46	122.08
C(13)	- C(14)	1.3922(13)	1.3930	-0.0003	0.0006	0.0005	0.0078(4)	0.0076(3)	0.0002(5)	0.0141	133.14	55.15	63.02
C(15)	- C(16)	1.3905(14)	1.3913	-0.0007	-0.0001	0.0005	0.0060(3)	0.0063(3)	0.0003(4)	0.0173	138.12	103.86	51.45
C(15)	- C(20)	1.4069(12)	1.4079	0	0	-0.0010	0.0066(3)	0.0060(3)	0.0006(4)	0.0245	94.17	102.13	167.16
C(16)	- C(17)	1.4024(12)	1.4032	-0.0006	-0.0001	-0.0005	0.0145(3)	0.0148(4)	0.0003(5)	0.0173	144.13	117.29	111.40
C(17)	- C(18)	1.3951(13)	1.3962	0	0	-0.0010	0.0127(4)	0.0131(4)	0.0004(5)	0.0200	93.98	102.56	166.81
C(18)	- C(19)	1.3997(14)	1.4005	0.0007	0.0001	-0.0005	0.0073(4)	0.0056(3)	0.0017(5)	0.0412	42.00	75.97	128.58
C(19)	- C(20)	1.3923(11)	1.3931	0.0006	0.0001	0.0005	0.0092(3)	0.0085(3)	0.0007(4)	0.0265	36.02	62.42	68.74

Sqrt(Sum(DelIJ**2)/Nrb) = 0.0006

# - Indicates bonds exceeding the 5.0 sigma test level

"t1n " PLATON-ADP-Anal Page 16

===

Test Matrix for Rigid-Body Vibrations - /Del(A,B)/ = /Z(A,B)**2 - Z(B,A)**2/ Should be Near Zero (Acta Cryst. A34, 1978, 828)

===

Atom-Atom		1	2	3	4	5	6	7	8	9	10	11	12	13	14	15	16	17	18	19	20
1 C(1)	-	0	1	0	0	0	1	0	0	0	0	0	0	0	0	0	1	0	0	1	0
2 C(2)	-	3	0	1	0	1	0	0	0	0	0	0	0	0	0	1	1	1	1	0	0
3 C(3)	-	-2	2	0	-1	1	1	1	-1	0	1	0	0	0	0	0	0	1	0	0	0
4 C(4)	-	3	4	-1	0	0	0	1	0	1	1	0	0	0	1	0	1	1	1	0	0
5 C(5)	-	4	4	2	-1	0	-1	1	0	1	1	0	0	0	1	0	0	2	2	2	1
6 C(6)	-	4	4	3	2	-1	0	0	0	0	1	0	0	1	0	1	1	1	1	1	0
7 C(7)	-	4	3	2	3	2	-1	0	0	0	0	1	0	1	0	0	0	2	2	2	0
8 C(8)	-	2	-2	-1	2	3	2	-1	0	0	0	1	1	0	0	1	1	2	1	1	0
9 C(9)	-	-2	2	2	4	5	5	4	3	0	0	1	0	0	0	0	1	1	0	0	0

Atom		1	2	3	4	5	6	7	8	9	10	11	12	13	14	15	16	17	18	19	20
10 C(10)	-	3	4	4	4	6	6	5	4	-1	0	0	1	1	1	0	0	1	1	1	1
11 C(11)	-	4	4	5	6	7	7	6	5	2	-1	0	0	0	0	1	0	2	2	1	1
12 C(12)	-	4	4	5	6	7	7	6	5	3	2	-1	0	0	0	0	1	2	1	1	1
13 C(13)	-	4	3	4	5	6	6	4	4	2	3	2	-1	0	0	1	1	1	1	1	1
14 C(14)	-	2	-2	3	4	5	5	4	2	-1	2	3	2	-1	0	1	1	1	1	0	0
15 C(15)	-	-2	2	2	4	5	5	4	3	2	4	5	5	4	3	0	0	1	0	0	-1
16 C(16)	-	3	4	4	5	6	6	5	4	4	5	6	6	5	4	-1	0	0	1	0	1
17 C(17)	-	4	4	5	6	7	7	6	5	5	6	7	7	6	5	2	-1	0	0	0	1
18 C(18)	-	4	4	5	6	7	7	6	5	5	6	7	7	6	5	3	2	-1	0	-2	1
19 C(19)	-	4	3	4	5	6	6	5	4	4	5	6	6	5	4	2	3	2	-1	0	-1
20 C(20)	-	2	-2	3	4	5	5	4	2	3	4	5	5	4	2	-1	2	3	2	-1	0

Remarks

- Upper Triangle Entries Represent /Del(A,B)/*1000 Values

- Lower Triangle Entries Represent Distances (A-B) Angstrom

- Negative Entries Indicate Bonded Atoms

===

DMANH^{+}×2Cl^{-}×$H_5O_2^{+}$

===

Rigid-Body Model Libration Corrections for Bond Distances and "Hirshfeld Rigid-Bond" Test (Acta Cryst., 1976, A32, 239-244)

===

Bond		Bond Distance		Components of the Correction			MSDA from U(obs) Vibration Along the Interatomic Bond				Angle with Lib. Axes		
Atom(I)	Atom(J)	Obsd	Calcd	Del(L)	Del(M)	Del(N)	I to J	J to I	Difference	Sqrt(Diff)	L(1)	L(2)	L(3)
N1	- C1	1.458(3)	1.4595	-0.0014	0.0004	-0.0001	0.0133(7)	0.0124(10)	0.0009(12)	0.0300	166.34	76.72	93.23
N1	- C11	1.484(3)	1.4866	0.0006	-0.0012	0.0028	0.0162(7)	0.0174(12)	0.0012(14)	0.0346	75.37	108.08	23.63
N1	- C12	1.474(4)	1.4767	0.0007	-0.0015	-0.0025	0.0131(7)	0.0113(15)	0.0018(17)	0.0424	73.89	130.91	134.65
N2	- C8	1.473(3)	1.4743	-0.0014	0.0003	0.0001	0.0129(7)	0.0136(10)	0.0007(12)	0.0265	168.91	78.93	89.45
N2	- C13	1.487(3)	1.4896	0.0003	0.0010	-0.0028	0.0102(7)	0.0121(13)	0.0019(15)	0.0436	66.10	79.44	153.57
N2	- C14	1.482(3)	1.4848	0.0005	0.0011	0.0025	0.0127(7)	0.0118(12)	0.0009(14)	0.0300	60.04	61.78	43.45
C1	- C2	1.376(3)	1.3784	-0.0005	-0.0021	0	0.0082(10)	0.0098(12)	0.0016(16)	0.0400	134.12	135.58	85.60
C1	- C9	1.432(3)	1.4349	-0.0009	0.0026	0	0.0124(10)	0.0112(10)	0.0012(14)	0.0346	105.27	17.16	97.70
C2	- C3	1.414(3)	1.4153	-0.0014	0.0004	-0.0001	0.0162(12)	0.0192(13)	0.0030(17)	0.0548	166.89	77.31	93.19
C3	- C4	1.375(3)	1.3771	-0.0009	0.0025	0	0.0178(13)	0.0201(11)	0.0023(17)	0.0480	106.91	18.74	97.71
C4	- C10	1.422(3)	1.4239	0.0005	0.0022	0.0001	0.0132(11)	0.0103(11)	0.0029(16)	0.0539	47.52	42.77	93.93
C5	- C6	1.375(3)	1.3772	0.0004	0.0021	0.0001	0.0171(11)	0.0168(12)	0.0003(17)	0.0173	48.56	41.67	93.70
C5	- C10	1.418(3)	1.4203	0.0009	-0.0025	0	0.0136(11)	0.0149(11)	0.0013(16)	0.0361	72.58	161.00	82.76
C6	- C7	1.415(3)	1.4167	0.0014	-0.0003	0	0.0155(12)	0.0165(12)	0.0010(17)	0.0316	11.77	101.67	88.03
C7	- C8	1.373(3)	1.3759	0.0009	-0.0024	-0.0001	0.0101(12)	0.0097(10)	0.0004(16)	0.0200	71.63	160.71	84.15
C8	- C9	1.426(3)	1.4278	-0.0004	-0.0023	0	0.0102(10)	0.0103(10)	0.0001(14)	0.0100	129.45	139.94	84.29
C9	- C10	1.432(3)	1.4332	-0.0014	0.0004	0	0.0132(10)	0.0117(11)	0.0015(15)	0.0387	167.38	77.67	92.52

Sqrt(Sum(DelIJ**2)/Nrb) = 0.0016

# - Indicates bonds exceeding the 5.0 sigma test level

===

Test Matrix for Rigid-Body Vibrations - /Del(A,B)/ = /Z(A,B)**2 - Z(B,A)**2/ Should be Near Zero (Acta Cryst. A34, 1978, 828)

===

Atom-Atom		1	2	3	4	5	6	7	8	9	10	11	12	13	14	15	16
1 N1	-	0	1	-1	2	2	1	1	0	1	1	1	1	-1	-2	2	1
2 N2	-	3	0	1	1	3	1	2	1	1	-1	2	1	4	5	-2	-1
3 C1	-	-1	3	0	-2	5	2	1	2	2	1	-1	0	1	1	6	5

4	C2	-	2	4	-1	0	-3	1	2	2	2	1	2	1	2	1	4	4
5	C3	-	4	5	2	-1	0	-2	0	0	1	1	1	0	2	2	2	3
6	C4	-	4	5	3	2	-1	0	2	1	1	0	0	-3	0	1	4	4
7	C5	-	5	4	4	4	4	2	0	0	1	1	0	-1	3	5	2	1
8	C6	-	5	4	4	5	5	4	-1	0	-1	0	0	0	5	8	4	2
9	C7	-	4	2	4	5	5	4	2	-1	0	0	1	2	6	9	4	0
10	C8	-	3	-1	3	4	4	4	3	2	-1	0	0	3	5	7	1	0
11	C9	-	2	2	-1	2	3	2	2	3	2	-1	0	-1	2	3	2	3
12	C10	-	4	4	2	3	2	-1	-1	2	3	2	-1	0	2	2	5	5
13	C11	-	-1	3	2	3	4	5	6	6	5	4	3	5	0	2	0	4
14	C12	-	-1	4	2	3	4	5	6	6	5	4	4	5	2	0	8	1
15	C13	-	3	-1	4	5	6	6	5	4	3	2	3	5	5	4	0	4
16	C14	-	4	-1	4	5	6	6	5	4	3	2	4	5	4	5	2	0

Remarks

- Upper Triangle Entries Represent /Del(A,B)/*1000 Values

- Lower Triangle Entries Represent Distances (A-B) Angstrom
- Negative Entries Indicate Bonded Atoms
